# The prevalence, metabolic disturbances and clinical correlates of recent suicide attempts in Chinese inpatients with major depressive disorder

**DOI:** 10.1186/s12888-019-2131-6

**Published:** 2019-05-10

**Authors:** Yue-Jiao Ma, Dong-Fang Wang, Ming Yuan, Xiao-Jie Zhang, Jiang Long, Shu-Bao Chen, Qiu-Xia Wu, Xu-Yi Wang, Marguerite Patel, Christopher D. Verrico, Tie-Qiao Liu, Xiang-Yang Zhang

**Affiliations:** 1 Department of Psychiatry, The Second Xiangya Hospital, Central South University, The China National Clinical Research Center for Mental Health Disorders, Chinese National Technology Institute of Psychiatry, Key Laboratory of Psychiatry and Mental Health of Hunan Province, No. 139, Middle Renmin Road, Changsha, Hunan 410011 People’s Republic of China; 2 Psychosomatic Health Institute of the Third Xiangya Hospital, Central South University, Changsha, Hunan People’s Republic of China; 3 Department of Psychiatry and Behavioral Sciences, Baylor College of Medicine, Houston, TX USA; 4 Institute of Psychology, Chinese Academy of Sciences, Beijing, China; 5 Department of Psychiatry and Behavioral Sciences, The University of Texas Health Science Center at Houston, 1941 East Road, Houston, TX 77054 USA

**Keywords:** Depression, Suicide attempt, Prevalence, Clinical correlates, Metabolic disturbance

## Abstract

**Background:**

Metabolic disturbances have been correlated with suicidality, but little is known about the association between suicide risk and metabolic disturbances among individuals with depression. This study was to evaluate the prevalence and clinical correlations, especially cardio-metabolic associated factors of recent suicide attempts in Chinese patients with major depressive disorder (MDD).

**Methods:**

A total of 288 MDD inpatients were recruited. Their clinical and demographic data together with plasma glucose, lipid and thyroid function parameters were collected. Self-Rating Depression Scale (SDS), Self-Rating Anxiety Scale (SAS) and Eysenck Personality Questionnaire (EPQ) were rated for most of the patients.

**Results:**

Of these MDD inpatients, 20.14% had attempted suicide during the past 1 month. Compared to those who had not attempted suicide, the suicide attempters had a significantly longer duration of illness, lower low-density lipoprotein (LDL) cholesterol, lower total cholesterol, and more psychotic symptoms. However, all these significant results did not survive after the bonferroni correction (all *p* > 0.05). A logistic regression analysis indicated that suicide attempts were associated with the lower total cholesterol and more psychotic symptoms.

**Conclusions:**

Our findings support the hypothesis of the association of low plasma cholesterol level and recent suicidal attempts in patients with MDD.

## Introduction

Suicide is a global phenomenon and accounts for 1.4% of all deaths worldwide, making it the 17th leading cause of mortality [1]. A previous study reported that 77% of individuals who had committed suicide had a mental disorder at the time of death, primarily depression (63%) [2]. Some previous studies reported that patients with depression had high rates of suicide ideation and attempt, which were associated factors for complete suicide [3–5]. Suicide has been considered as the most devastating outcome for patients with depression. However, the exact associated factors for suicide attempt in major depressive disorder (MDD) patients are still unclear [6]. Previous studies reported that the associated factors included male gender [7], unemployment [8], severe depressive pathology [9], psychotic symptoms [10, 11] and increased duration of depressive symptoms [12]. However, the results are inconsistent [6].

Recently, researchers have focused on identifying biomarkers that could be associated with suicidal behavior, such as thyroid hormone [13] and lipid dysfunction [14]. Especially, the association between lipid level and suicide behavior/attempt received great attention. However, the results have been inconsistent. For example, while low total cholesterol levels have been associated with suicide attempts in patients with depression [15–17], high serum total cholesterol levels have also been found to be associated with suicide in patients with depression [18–20]. Similarly, a recent study found associations between suicide and levels of both high density lipoprotein (HDL) and very low-density lipoprotein (VLDL) [21], while another study found no association [22]. In addition, recent studies have reported negative association between triglycerides and current suicidality [21, 23]. The heterogeneity of findings from these studies may arise from several factors, such as how suicidal behaviors were evaluated, as well as the populations being studied [24].

However, most previous studies focused on total cholesterol as a biomarker of interest, which might not be sufficient to display the relationship between suicide attempt and lipids. Hence, it is important to investigate the association between suicide attempt and lipid profile. The present study also included other blood markers, such as plasma glucose and thyroid hormone. Moreover, taking consideration about the duration from suicide attempt to blood sampling may impact the result, we only evaluated the suicide attempt in last month in this study. In addition, a recent study reported that suicide rates and the risk factors differed among countries [25, 26]. However, few studies that have investigated the prevalence of suicide attempts in Chinese people with depression revealed a wide range of prevalence (18.5 to 23.5%), with inconsistent associated factors [27, 28]. Thus, it is necessary to clarify this difference between different races. Therefore, the main aims of our study were to investigate the prevalence of recent suicide attempts in MDD inpatients, and the possible clinical and biological associated factors of suicide attempts in a Chinese Han inpatient population with MDD.

## Methods

### Sample

This was a cross-sectional naturalistic study conducted at the Mental Health Institute, Central South University Xiang-Ya Second Hospital, Changsha City, Hunan province, China. Two hundred and eighty-eight inpatients were recruited between June 01,2016 and May 01,2017. All patients met the following inclusion criteria: 1) 18 to 78 years old; 2) Han Chinese; 3) two psychiatrists with more than 10 years of clinical experience independently assessed the patient’s psychiatric history and made a diagnosis for the same patient according to the Diagnostic and Statistical Manual of Mental Disorders, fifth edition (DSM-V) criteria; 4) the ability to understand the meaning of each scale entry; 5) Patients who are not diagnosed with depressive episode of bipolar disorder or post-schizophrenia depression, and no co-morbidity with alcohol or substance use disorders or other psychotic disorders; and 6) no major medical abnormalities, including no central nervous system diseases, and no acute, unstable or life-threatening medical illnesses (e.g. cancer, infections).

The sample size was calculated using the formula, n = Z^2^p(1-p)/d^2^ [29]. Z was the degree of freedom at 95%, confidence interval being 1.96, d was desired marginal of errored, set to 0.05, p was the proportion of the population estimated to have a particular characteristic. According to a recent report in China, 23.5% of depressed patients had suicide attempt [28], This gave a sample estimate of 276 patients. However, selected sample size of 288 depressed patients was used based on the duration of the study.

The study protocol was approved by the Mental Health Institute of Central South University Xiang-Ya Second Hospital. All subjects provided signed, informed consent to participate in this study.

### Socio-demographic characteristics and clinical measures

On the first day of admission, a trained research staff conducted a detailed questionnaire survey of all patients to collect social-demographic data and clinical characteristics, including age of onset, first episode or recurrence, the total duration of illness from the onset of first episode, psychotic symptom, medication history and family history of mental illness. Suicidal attempts were defined as a seriously self-destructive act with the intention of ending one’s life but not resulting in death and excluded suicidal idea or all self-injury behaviors without the ‘intention’ to die, as well as the low-lethal self-harm behaviors [30]. We only evaluated the suicide attempts in the last month. Detailed information was collected including number of suicide attempts, and the exact methods for each attempt. Additional information was collected from medical records and collateral resources. If data was missing or ambiguous, the research staff conducted an addition visit with family members or the patient’s clinical team to complete the history.

The Self-Rating Depression Scale (SDS) and Self-Rating Anxiety Scale (SAS) were used to describe mood status. Eysenck Personality Questionnaire (EPQ) was used to assess the personality traits. The EPQ includes four subscales: internal and external tilt scale (E), emotional scale (N), psychological metamorphosis scale (P, also known as mental quality) and efficacy scale (L). All of the scales used in this study have Chinese version, have good reliability and validity, and also have been widely used in China.

### Blood samples

Blood samples were collected between 6 and 7 am following an overnight fast on the day after admission. All samples were sent to the laboratory center of the hospital immediately and measured before 11 am on the same day. Plasma glucose, serum total cholesterol, triglycerides, HDL cholesterol (HDL-c) and LDL cholesterol (LDL-c) were measured on an ARCHITECT c8000 System (Abbott Laboratories, Irving, TX, USA). The serum levels of thyroid hormone including thyroid stimulating hormone (TSH), free triiodothyronine (FT3) and free tetraiodothyronine (FT4) were analyzed using ARCHITECT Immulite 2000 SR analyzer (Abbott, Longford, Ireland) by a Chemiluminescent Microparticle immunoassay (CMIA).

### Statistical analysis

The majority of the variables were not normally distributed in suicide attempters (Kolmogorov–Smirnov one-sample test), and only part of the variables were normally distributed in non-attempters. All demographic and clinical data were compared between participants with and without suicide attempts, using the Mann–Whitney nonparametric test for continuous variables and chi-square test for categorical variables.

Prevalence of suicide attempts was described with percentage and analyzed by chi-square test. Odds ratios (OR) derived from logistic regression analyses was used to compare suicide attempters to non-attempters after adjusting for the related variables. Correlations among demographic, clinical variables and biological parameters were examined by Spearman correlation coefficients. A binary logistic regression was used to analyze the parameters that were most strongly associated with suicide attempts. The statistical analyses were performed using SPSS22.0 for Windows. All statistical tests were two-tailed and the significance level was set at *P* < 0.05. Bonferroni corrections were used to account for multiple testing, which were performed through multiplying the unadjusted *P* value by the number of comparisons made to obtain the adjusted P value, compare it with set P value (0.05).

## Result

A total of 288 MDD patients were enrolled, with the mean age 39.37 ± 14.05 years. 58 patients reported at least one suicide attempt history in the past 1 month, and the overall suicide attempt rate was 20.14%. Suicide attempt rate was similar between female (20.21%) and male (20.00%) patients (Χ^2^ = 0.002, df = 1, *p* = 0.99). Methods of attempt included drug overdose (*n* = 23), self-inflicted knife wound (*n* = 19), jumping from heights (*n* = 6), attempted drowning (*n* = 5), hanging (n = 5), tongue biting (n = 2), ingestion of toxins (n = 2), electrocution (n = 2), and intentional motor vehicle accident (n = 1). 19 patients (32.76%) had psychotic features at time of attempt, including delusions of guilt or unworthiness (n = 6), delusions of persecution (n = 6), auditory hallucinations (*n* = 4), delusions of reference (n = 5), delusions of control (n = 1), disturbance of body-image sensorial synthesis (n = 1), and delusions of hypochondria (n = 1).

Table 1 shows descriptive statistics of socio-demographic data and clinical variables in suicide attempters and non-attempters*.* Compared to non-attempters, the attempters had a longer duration of illness (Z = -2.10, *p* = 0.04), higher rate of recurrent depression (Χ^2^ = 3.86, *p* = 0.05), more psychotic symptoms (Χ^2^ = 4.67, *p* = 0.03), lower LDL cholesterol (Z = -1.97, p = 0.05) and lower total cholesterol (Z = -2.17, p = 0.03). There were no significant differences between the groups after Bonferroni correction.

**Table 1:** Characteristics of depressed patients with and without suicide attempts

	All patients (*n* = 288)	Suicide attempt (*n* = 58)	Non- suicide attempt (*n* = 230)	Z/χ2	P
Socio-demographic and clinical characteristics(med ± IQR)					
Age (years)	41(24)	39(27)	42(23)	−0.77	0.44
Female (%)	188(65.28%)	38(65.52%)	150(65.22%)	0.002	0.99
Unmarried (%)	96(33.33%)	19(32.76%)	77(33.48%)	0.01	0.91
Unemployment (%)	101(35.07%)	26(44.83%)	75(32.61%)	3.04	0.08
Education(years)	12(7)	12(5)	12(7)	−1.74	0.08
Recurrence (%)	171(19.38%)	41(70.69%)	130(56.52%)	3.86	0.05
Age of onset(years)	36.00(26)	31(30.25)	37(24)	−1.65	0.10
Duration of illness(month)	12.5(57)	32.5(60.25)	12.(45)	−2.10	0.04
Family history of mood disorder (%)	42 (14.58%)	7 (12.07%)	35 (15.22%)	0.30	0.82
Drug-naïve (%)	126 (43.75%)	20 (34.48%)	106 (46.09%)	2.54	0.11
Psychotic symptom (%)	64 (22.22%)	19(32.76%)	45 (19.57%)	4.67	0.03
Diabetes (%)	15(5.20%)	4(6.90%)	11(4.78%)	0.41	0.52
High blood pressure (%)	36(12.50%)	6(10.34%)	30(13.04%)	0.32	0.57
Thyroid disease (%)	13(4.51%)	4(6.90%)	9(3.91%)	0.96	0.33
Scale assessment(med ± IQR)					
SAS	58(16)	58.5 (15)	56 (16)	−0.63	0.53
SDS	67(14.5)	70.5(13.25)	66(14)	−1.70	0.09
EPQ—E	8(5)	9(4.75)	8(6)	−0.53	0.60
EPQ—N	15(7)	15(6)	15(7)	−0.16	0.87
EPQ—P	8(4)	8(5.8)	8(5)	−1.14	0.25
EPQ—L	11(4)	10.5(5.5)	11(4)	−1.50	0.13
Biological indicators(med ± IQR)					
Fasting plasma glucose (mmol/L)	4.56(0.71)	4.62(0.88)	4.55(0.69)	−0.12	0.91
Triglycerides (mmol/L)	1.17(0.78)	1.27(0.86)	1.14(0.74)	−0.91	0.37
HDL cholesterol (mmol/L)	1.27(0.44)	1.25(0.41)	1.27(0.44)	−0.97	0.33
LDL cholesterol (mmol/L)	2.51(0.92)	2.38(1.07)	2.58(0.90)	−1.97	0.05
Total Cholesterol (mmol/L)	4.18(1.20)	4.08(1.04)	4.28(1.18)	−2.17	0.03
HD/CH (mmol/L)	0.30(0.10)	0.31(0.10)	0.30(0.10)	−0.35	0.72
FT3 (pmol/L)	4.60(1.12)	4.61(1.12)	4.59(1.14)	−0.18	0.86
FT4 (pmol/L)	14.84(3.56)	14.35(4.21)	14.93(3.52)	−0.97	0.33
TSH (mIU/L)	1.51(1.26)	1.60 (1.65)	1.48(1.24)	−1.48	0.14

Note: *HDL* High density lipoprotein, *LDL* Low density lipoprotein, *HD/CH* HDL cholesterol/total cholesterol, *FT3* Free triiodothyronine, *FT4* Free tetraiodothyronine, *TSH* Thyroid stimulating hormone, *SDS* Self-Rating Depression Scale, *SAS* Self-Rating Anxiety Scale, *EPQ* Eysenck Personality Questionnaire, *EPQ-E* Eysenck Personality Questionnaire internal and external tilt scale, *EPQ-N* Eysenck Personality Questionnaire emotional scale, *EPQ-P* Eysenck Personality Questionnaire psychological metamorphosis scale (P, also known as mental quality), *EPQ-L* Eysenck Personality Questionnaire efficacy scale

In Spearman correlation analysis, duration of illness (r = 0.12, p = 0.04), LDL cholesterol level (r = 0.17, p = 0.05) and total cholesterol level (r = − 0.13, p = 0.03) were significantly related to suicidal attempts. There were no significant correlations after Bonferroni correction (all *p* > 0.05).

In logistical regression analysis, psychotic symptoms (standardized β = 0.88, *p* = 0.01) and lower total cholesterol (TC) (standardized β = − 1.03, p = 0.03) were significantly associated with having a recent suicide attempt (Table 2).

**Table 2:** Predictors of suicide attempt within a linear regression model

	Coefficients					95.0% Confidence Interval for β	
	β	Std. Error	Wald	p value	Exp (β)	Lower Bound	Upper Bound
Duration of illness	0.004	0.002	2.41	0.12	1.00	0.99	1.01
Recurrence	0.43	0.37	1.36	0.24	1.54	0.75	3.15
Psychotic symptom	0.88	0.34	6.59	0.01	2.40	1.23	4.69
low density lipoprotein cholesterol	0.74	0.54	1.83	0.18	2.09	0.72	6.05
Total Cholesterol	−1.03	0.46	4.92	0.03	0.36	0.15	0.89

## Discussion

This was an exploratory cross-sectional clinical study, examining the socio-demographic, clinical characteristics and biochemical parameters associated with suicide attempts in Chinese inpatient subjects with a diagnosis of major depressive disorder. Our study showed that the prevalence of patients with recent suicide attempts in this population was 20.14%, and the significant associated factors predictive of recent suicide attempts are more psychotic symptoms and lower total cholesterol.

The 20.14% prevalence in our study was within the range of 18.5 and 23.5% found in two multi-center studies among patients with major depressive disorder in China [27, 28] . Studies from other countries reported rates of suicide attempts in depression including 16.9% in upper northern Thailand [31] and 19.8% in Korea [4]. In France, 33.7% patients with MDD and bipolar major depression had reported lifetime suicide attempts [32]. Thus, the rate found in our study is similar to those reported in the other countries (16.9%~ 33.7%). The variability between studies may be related to differences in the definition of suicide attempt used and sample selection. Also, these results suggest that the rate of suicide attempts in depression may even vary within the same ethnic group (e.g. Chinese), possibly due to regional differences in social, cultural, economic contexts and in the access to psychiatric care.

Our study found that total cholesterol level was significantly lower in patients with suicide attempts during the past 1 month, a finding that is consistent with past work linking cholesterol levels to suicidal behavior/attempt [16, 33]. This has led to interest in using total cholesterol as a biomarker for suicidal risk in depressed patients. Interestingly, low cholesterol levels have been linked to suicidal behavior in other diagnoses. For example, schizophrenia patients who had attempted suicide were found to have lower total cholesterol when compared to those without suicide attempts [34], and another recent study found lower level of serum cholesterol in suicidal male patients with bipolar disorder [35]. In addition, lower cholesterol levels were detected in suicidal compared to non-suicidal patients with first episode of psychosis [36].

While these findings make a strong case for an association between low total cholesterol level and suicidal behavior, other studies have reported no association between serum cholesterol and suicide [37–41], or association in the opposite direction [20, 42]. The first possible explanation for these inconsistent results may be due to different time frames for suicide among different studies. For example, our study only included recent suicide attempts, while the others evaluated the lifetime suicide attempts [39]. Second, different definitions for suicide have been used in previous studies, including suicidal thoughts or ideation, plans, and death [20, 37, 42]. Third, in our current study we focused only on major depressive disorder, excluding those bipolar disorder [39], general population [20, 42] and other mental disorders [37, 38, 41], which are different from the previous studies. Fourth, other associated factors with suicide attempts were not included in most of previous studies, while our current study included several new clinical and psychological factors. Fifth, different methods to assess suicide attempts were used in previous studies. Finally, racial differences should also be considered.

There are several possible physiological explanations linking cholesterol levels to mood, one of which is via modulation of the serotonin (5HT) system. Animal studies have shown that lower cholesterol levels alter viscosity and functions of serotonin receptors and transporters [43, 44]. In monkeys, lower total serum cholesterol has been linked to lower central nervous system serotonin activity [45], and another study showed that low cholesterol levels had a negative impact on mood by a direct effect on the 5HT system, leading to increased depression, aggression, and suicide [46]. Cholesterol levels have also been linked to alterations in levels of serotonin metabolite 5-hydroxyindoleacetic acid (5-HIAA) in the cerebrospinal fluid (CSF), which is associated with suicide risk [47]. In line with these data, reduced cholesterol levels and reduced platelet serotonin concentrations were also detected in suicidal compared to non-suicidal male patients with first episode of psychosis [36]. Taken together, these studies suggest that low TC may be a potential associated factor for suicide through its influence on 5HT system.

Another secondary finding was the link between psychotic features and increased risk of suicide attempts in depressed patients, which is consistent with previous studies [48, 49]. Studies have found that suicide risk is up to 4.04 times higher in patients with MDD with psychosis when compared to similar patients with non-psychotic major depression [50], especially during the acute episode of the illness [49]. However, a few studies showed no differences in suicidal behaviors between patients with psychotic major depression (PMD) and non-psychotic major depression (NMD) [51, 52]. The heterogeneity of these results may be due to the different age period of sample [51], or different research design [52]. It is of interest to speculate the underlying mechanisms for this link. For example, psychosis may reduce thought organization, increase paranoia, or reduce mental flexibility. However, the exact reasons for the link between psychotic features and increased risk of suicide attempts in depression deserve further investigation.

The first strength of this current study was that we focused on recent suicide attempts, which made the correlation results more accurate and avoided the impact of the duration from suicide attempt to blood sampling. Another strength was that we assessed the objective blood markers for suicide attempt rather than only the clinical characteristics, which few studied examined in the Chinese Han population before. There are several limitations in our current study. First, this was a cross-sectional study, which cannot establish the causal relationship between suicide attempts and associated factors in depressed patients. Second, we had comparatively small sample size in this study, and our sample was limited to the depressed inpatients only in the Changsha area and cannot be generalized to the outpatient or community patients in other regions in China. Hence, our results need to be validated in a larger sample and wider areas in the future. Third, we collected information through a research interview with patients, family members, and clinicians rather than using a structured instrument, which may have introduced bias during the collection phase. In addition, data was only collected for suicide attempts and not for suicidal thoughts or ideation, which may have led us to miss elevated suicidality in certain patients. Fourth, suicidal behavior was not able to be divided into the smaller subgroups, such as violent and non-violent suicide subgroups due to the limited sample size. This limitation is important as previous studies have shown that total cholesterol was significantly different between MDD patients with violent and non-violent suicides [21]. Fifth, we did not collect data on body mass index (BMI) and dietary, which may have an impact to metabolic parameters [53] and suicide attempt [54], although previous researches revealed not significant in studied group [16, 55]. Finally, Suicide related behaviors are complex and multifaceted phenomenon, which may associated with many other clinical and unknown biological factors. However, the factors that we explored are not inclusive of all those studied in the literature.

## Conclusion

In summary, the present study revealed a prevalence of a recent suicide attempts (20.14%) in depressed inpatients which was consistent with previous findings of 18.5–23.5% suicide attempt rate in patients with major depressive disorder in the Chinese population. Compared to the non-attempters, patients who made suicide attempts had longer duration of illness, more psychotic symptoms, lower LDL cholesterol and lower total cholesterol. However, owing to the limitations of a relatively limited sample size and cross-sectional design, our findings should be interpreted with caution and the results need be confirmed in a larger sample size using a longitudinal design in the future.

## Abbreviations

CSF: Cerebrospinal fluid
EPQ: Eysenck Personality Questionnaire
EPQ-E: EPQ internal and external tilt scale
EPQ-L: EPQ efficacy scale
EPQ-N: EPQ emotional scale
EPQ-P: EPQ psychological metamorphosis scale
FT3: Free triiodothyronine
FT4: Free tetraiodothyronine
HD/CH: HDL cholesterol/total cholesterol
HDL: High density lipoprotein
LDL: Low-density lipoprotein
MDD: Major depressive disorder
NMD: Non-psychotic major depression
PMD: Psychotic major depression
SAS: Self-Rating Anxiety Scale
SDS: Self-Rating Depression Scale
TC: Total cholesterol
TSH: Thyroid stimulating hormone
VLDL: Very low-density lipoprotein
